# Uric acid enhances alteplase-mediated thrombolysis as an antioxidant

**DOI:** 10.1038/s41598-018-34220-1

**Published:** 2018-10-26

**Authors:** Kiyoshi Kikuchi, Kentaro Setoyama, Eiichiro Tanaka, Shotaro Otsuka, Takuto Terashi, Kazuki Nakanishi, Seiya Takada, Harutoshi Sakakima, Sumate Ampawong, Ko-ichi Kawahara, Tomoka Nagasato, Kazuya Hosokawa, Yoichiro Harada, Mika Yamamoto, Chinatsu Kamikokuryo, Ryoji Kiyama, Motohiro Morioka, Takashi Ito, Ikuro Maruyama, Salunya Tancharoen

**Affiliations:** 1 Division of Brain Science, Department of Physiology, Kurume University School of Medicine, Kurume, Japan; 2 Department of Neurosurgery, Kurume University School of Medicine, Kurume, Japan; 3 Department of Systems Biology in Thromboregulation, Kagoshima University Graduate School of Medical and Dental Science, Kagoshima, Japan; 4 Department of Pharmacology, Faculty of Dentistry, Mahidol University, Bangkok, Thailand; 5 Natural Science Center for Research and Education, Division of Laboratory Animal Science, Kagoshima University, Kagoshima, Japan; 6 Course of Physical Therapy, School of Health Sciences, Faculty of Medicine, Kagoshima University, Kagoshima, Japan; 7 Department of Tropical Pathology, Faculty of Tropical Medicine, (S.A.), Mahidol University, Bangkok, Thailand; 8 Laboratory of Functional Foods, Department of Biomedical Engineering, Osaka Institute of Technology, Osaka, Japan; 9 Research Institute, Fujimori Kogyo Co., Yokohama, Kanagawa Japan; 10 Department of Emergency and Critical Care Medicine, Kagoshima University Graduate School of Medical and Dental Science, Kagoshima, Japan; 11 School of Health Sciences, Faculty of Medicine, Kagoshima University, Kagoshima, Japan

## Abstract

Uric acid (UA) therapy may prevent early ischemic worsening after acute stroke in thrombolysis patients. The aim of this study was to examine the influence of UA on the thrombolytic efficacy of alteplase in human blood samples by measuring thrombolysis under flow conditions using a newly developed microchip-based flow-chamber assay. Human blood samples from healthy volunteers were exposed to UA, alteplase, or a combination of UA and alteplase. Whole blood and platelet-rich plasma were perfused over a collagen- and thromboplastin-coated microchip, and capillary occlusion was monitored with a video microscope and flow-pressure sensor. The area under the curve (extent of thrombogenesis or thrombolysis) at 30 minutes was 92% lower in the UA–alteplase-treated group compared with the alteplase-treated group. D-dimers were measured to evaluate these effects in human platelet-poor plasma samples. Although hydrogen peroxide significantly decreased the elevation of D-dimers by alteplase, UA significantly inhibited the effect of hydrogen peroxide. Meanwhile, rat models of thromboembolic cerebral ischemia were treated with either alteplase or UA–alteplase combination therapy. Compared with alteplase alone, the combination therapy reduced the infarct volume and inhibited haemorrhagic transformation. UA enhances alteplase-mediated thrombolysis, potentially by preventing oxidative stress, which inhibits fibrinolysis by alteplase in thrombi.

## Introduction

Acute ischemic stroke (AIS) is a major cause of death worldwide and causes various severe disorders^1^. Alteplase and endovascular mechanical thrombectomy are currently the two major treatments for AIS^2^. In the future, endovascular mechanical thrombectomy may become the first choice of treatment. However, currently it is common to administer alteplase before endovascular mechanical thrombectomy. Alteplase is the most effective and frequently used recombinant tissue plasminogen activator for thrombolysis in patients with AIS^3^. Tissue plasminogen activator (tPA) catalyses plasminogen to plasmin if alteplase is administered via the intravenous route, which promotes endogenous fibrinolysis and vessel recanalization^4^.

The URICO-ICTUS trial (Efficacy Study of Combined Treatment with Uric Acid and rtPA in Acute Ischemic Stroke) showed that uric acid (UA) therapy significantly reduced the incidence of early ischemic worsening compared with placebo in patients treated with alteplase within 4.5 hours of AIS onset^5^. In experimental stroke models, UA therapy reduced brain damage and acted synergistically with alteplase in thromboembolic models^6^. Oxidative stress has been shown to induce atherogenesis, thrombosis, and atherosclerosis, which subsequently caused brain injury^7^. Therefore, antioxidants, such as UA, may have neurovascular protective effects by pleiotropic mechanisms^8^.

Oxidative stress may induce plasminogen activator inhibitor-1 (PAI-1)^9,10^. Therefore, UA may affect the blood itself, and not act solely as a neurovascular protectant. Current *in vitro* assays of fibrinolytic reactions, such as clot–lysis tests, thromboelastography, and rotational thromboelastometry, are generally performed in the absence of blood flow; this limits their relevance to pathologic arterial thrombosis or physiological haemostasis^11,12^. To overcome the limitations associated with animal models and static *in vitro* assays for assessing fibrinolysis, Hosokawa *et al*. speculated that evaluating fibrin-rich platelet thrombus formation under shear flow could be a useful model for studying thrombolytic processes in the arterial circulation^13^.

The aim of our study was to examine the mechanism by which UA promotes alteplase-mediated thrombolysis *in vitro* in human blood donated by healthy volunteers. We used the newly developed Total Thrombus-formation Analysis System (T-TAS®, Fujimori Kogyo Co., Ltd., Tokyo, Japan) to quantify thrombolysis in whole blood and platelet-rich plasma (PRP) exposed to UA, alteplase, or UA and alteplase. We also measured the concentration of D-dimer, a fibrin degradation product, in PRP sump solutions collected after the T-TAS assay to determine whether UA-alteplase combination therapy inhibits thrombogenesis or promotes thrombolysis. Next, we measured the concentration of D-dimer in platelet-poor plasma (PPP), and examined the influence of hydrogen peroxide (H_2_O_2_) on alteplase-induced thrombolysis. Finally, we also examined the influence of UA on H_2_O_2_-inhibition of thrombolysis in PPP.

## Results

### Characteristics of blood samples obtained from healthy volunteers

The mean erythrocyte, leukocyte, and platelet counts of whole blood and PRP samples are shown in Table 1. These lay within the normal ranges for healthy Japanese individuals.

**Table 1:** Erythrocyte, leukocyte, and platelet counts in whole blood and platelet-rich plasma used in the Total Thrombus-formation Analysis System assay.

	Whole blood	Platelet-rich plasma
Erythrocyte count (×10^6^/µl)	5.10 ± 0.37	0.02 ± 0.01
Leukocyte count (×10^3^/µl)	6.21 ± 1.11	0.01 ± 0.01
Platelet count (×10^3^/µl)	258 ± 53	406 ± 78

Values are shown as mean ± standard deviation.

### UA Enhances Alteplase-mediated Thrombolysis in Human Whole Blood

**Figure 1:**
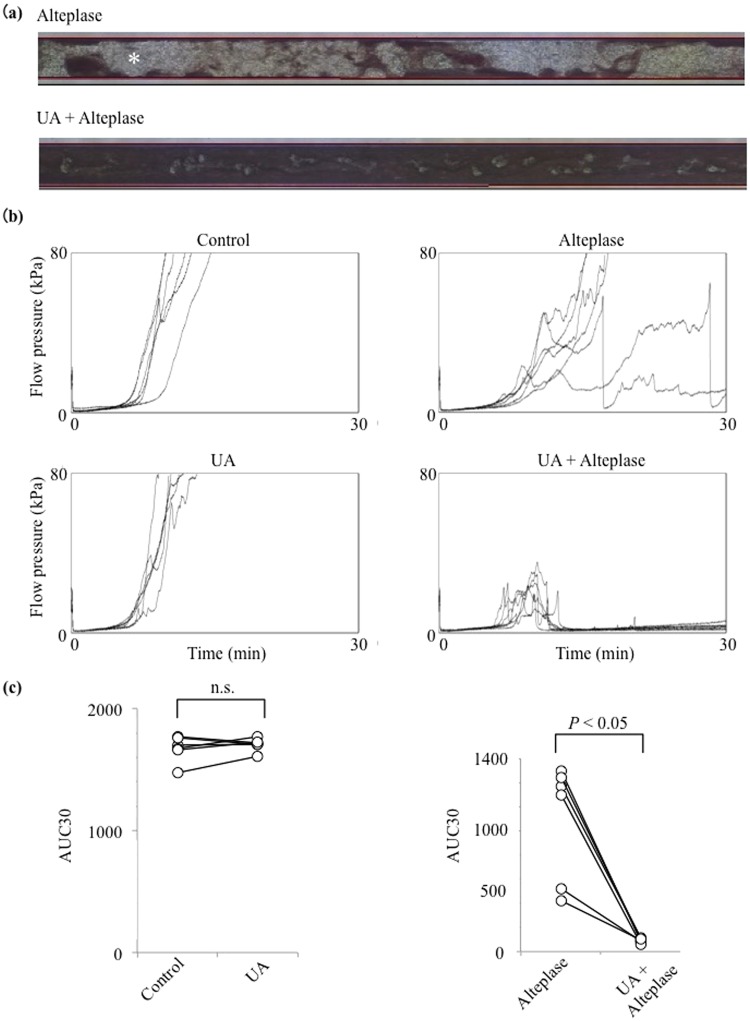
Effect of UA on alteplase-induced thrombolysis under flow conditions in whole blood. (**a**) Representative still videomicroscopy images of thrombogenesis and thrombolysis over 15 to 16 min in samples exposed to alteplase or UA–alteplase in whole blood. The asterisk (white area) indicates thrombi. (**b**) Flow–pressure curves in the control, UA, alteplase, and UA–alteplase groups in whole blood (n = 6). (**c**) AUC30 in the control, UA, alteplase, and UA–alteplase groups in whole blood (n = 6). Abbreviations: UA, uric acid; AUC30, area under the curve at 30 min; n.s., not significant.

To clarify the mechanism of the synergistic effects of UA in the URICO-ICTUS trial^5^, we evaluated whether UA enhances alteplase-mediated thrombolysis under flow conditions using the T-TAS in human whole blood. After perfusion had started, plentiful small white thrombi were observed adhering to the coated surface. Thrombus formation caused microcapillary occlusion in all control samples and UA samples (Fig. 1a). The thrombi gradually increased in size and merged with each other, leading to capillary occlusion in 9 to 13 min in the control and UA groups. In the alteplase, capillary occlusion occurred at 15 to 17 min. However, in the UA–alteplase group, the thrombi had dissolved within 13 min (Fig. 1b). Treatment with alteplase alone had a limited effect on thrombus firmness, but in the presence of UA, thrombus firmness diminished as evidenced by the frequent collapse of thrombi.

However, the perfused capillary was completely occluded in the four samples exposed to alteplase. In the alteplase non-responders (Supplementary Movie 1, left panel), thrombolysis as evidenced by reduced thrombus firmness and the frequent collapse of thrombi were observed in samples to which both UA and alteplase had been added (Supplementary Movie 1, right panel).

Microcapillary occlusion occurred in all control samples and the UA group and in four samples in the alteplase group but in none of the samples in the UA–alteplase group (Fig. 1b). We observed a characteristic periodic flow–pressure pattern that reflected the collapse of thrombi, consistent with the lack of microcapillary occlusion in the UA–alteplase group. The synergistic effect of UA–alteplase combination therapy was evaluated by calculating the AUC30, which was significantly lower in the UA–alteplase group (87.3 ± 19.0) than in the alteplase group (1091.5 ± 486.9) (*P* < 0.05) (Fig. 1c). There was no significant difference in the AUC30 between the control group (1672.2 ± 107.6) and UA group (1706.2 ± 51.7) (Fig. 1c). In conclusion, UA enhanced alteplase-mediated thrombolysis in human whole blood.

### UA Enhances Alteplase-mediated Thrombolysis in Human Plasma Components

**Figure 2:**
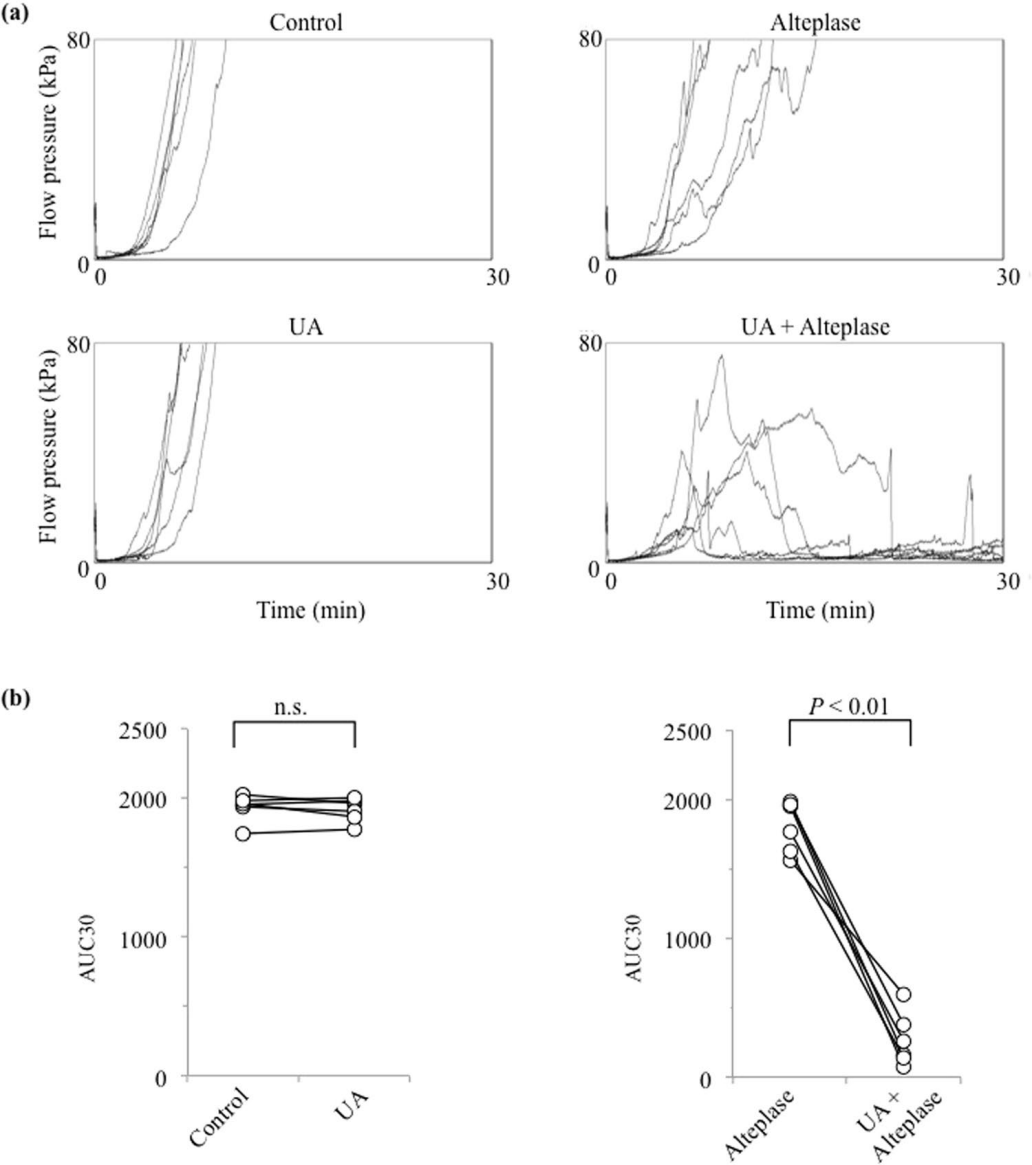
Effect of UA on alteplase-induced thrombolysis in PRP and PPP. (**a**) Flow–pressure curves in the control, UA, alteplase, and UA–alteplase groups in PRP under flow conditions (n = 6). (**b**) AUC30 in the control, UA, alteplase, and UA–alteplase groups in PRP. Abbreviations: AUC30, area under the curve at 30 min; PRP, platelet-rich plasma; n.s., not significant.

Which components of the whole blood were affected by UA is unclear. A comparison of whole blood and PRP samples revealed negligible numbers of erythrocytes and leukocytes in PRP samples (Table 1). However, the blood components affected by UA might be determined by comparing its effect between whole blood and PRP samples. Therefore, we examined the thrombolytic effects of alteplase, UA, and their combination under flow conditions using the T-TAS in PRP (Fig. 2). The synergistic effect of alteplase and UA was reflected by a significantly lower AUC30 in the UA–alteplase group (267.2 ± 191.0) compared with the alteplase group (1809.2 ± 186.3) (*P* < 0.01) (Fig. 2b). There was no significant difference in the AUC30 between the control (1934.2 ± 96.8) and UA groups (1917.7 ± 85.1) (Fig. 2b). In conclusion, the thrombolysis-enhancing effect of UA was also confirmed in PRP samples.

**Figure 3:**
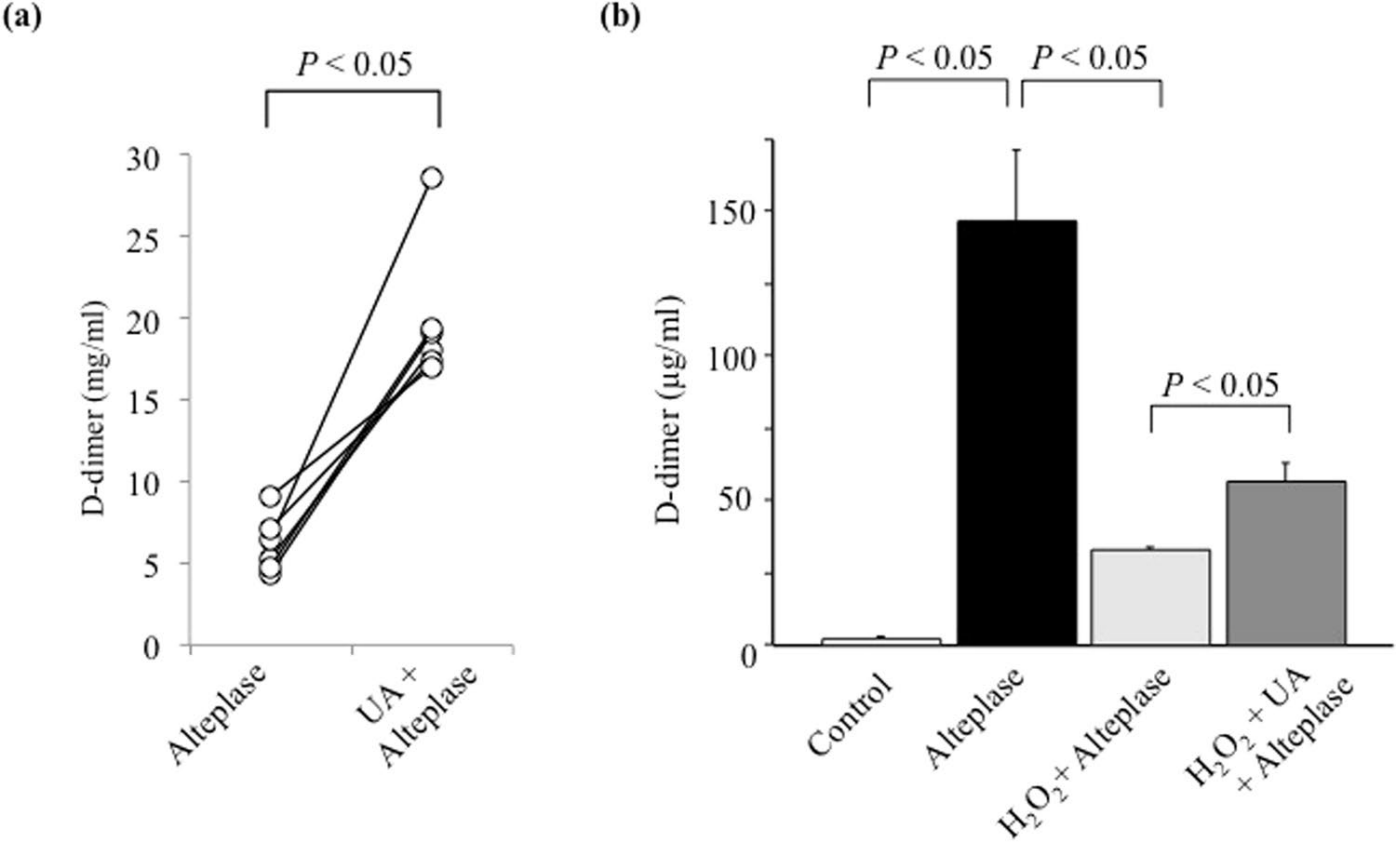
D-dimer in PRP sump solutions and PPP. (**a**) D-dimer concentration in sump solutions after measurement using the Total Thrombus-formation Analysis System in PRP in the alteplase and UA–alteplase groups (n = 6). (**b**) Measurement of D-dimer concentration to evaluate the effects of UA (10 mg/dL) on H_2_O_2_ (100 µM)-inhibited fibrinolysis by alteplase (100 IU/ml) in pooled PPP (n = 5). Error bars represent the standard deviation. Abbreviations: PRP, platelet-rich plasma; PPP, platelet-poor plasma; H_2_O_2_, hydrogen peroxide.

The thrombolysis-enhancing effect of UA was confirmed objectively and quantitatively in whole blood and PRP (Figs 1, 2). However, whether a low AUC on the T-TAS inhibits thrombogenesis or enhances thrombolysis remains unclear. We evaluated D-dimers in PRP sump solutions after measuring PRP samples using the T-TAS to confirm the thrombolysis-enhancing effect of UA in healthy human blood samples (Fig. 3a). The mean concentration of D-dimers in the PRP sump solutions collected after the T-TAS assay was significantly higher in the UA–alteplase group (19.9 ± 4.3 mg/ml) than in the alteplase alone group (6.2 ± 1.8 mg/ml) (*P* < 0.05) (Fig. 3a), demonstrating an inverse relationship between the AUC30 measured by the T-TAS and the D-dimer concentration. The D-dimer concentrations in all control samples and UA samples (100%) were <0.03 mg/ml, which is below the measurable range. These findings suggest that thrombolysis as evaluated by the T-TAS is strongly correlated with the D-dimer level.

The synergistic effect of UA–alteplase combination therapy was confirmed objectively by the AUC30 and quantitatively in both whole blood and PRP (Figs 1, 2, 3a). However, the effects of UA–alteplase combination therapy on platelets or plasma are unclear because both platelets and plasma were present in both whole blood and PRP samples. However, almost no platelets were present in PPP samples. Therefore, the blood components affected by UA might be clarified by comparing its effect separately in whole blood samples, PRP samples, and PPP samples. PPP samples could not be evaluated by the T-TAS assay because the capillaries did not become occluded in the T-TAS assay based on the lack of platelets observed. Therefore, we examined the effects of alteplase, UA, and their combination on fibrinolysis in PPP using a method other than the T-TAS (Fig. 3b).

The D-dimer concentration was significantly higher in PPP samples exposed to alteplase at 100 IU/ml than in controls (*P* < 0.05) (Fig. 3b). Moreover, in samples exposed to alteplase, the D-dimer concentration was significantly reduced by the addition of H_2_O_2_ (*P* < 0.05) (Fig. 3b). Nevertheless, in samples exposed to alteplase and H_2_O_2_, the D-dimer concentration was significantly increased by the addition of UA (*P* < 0.05) (Fig. 3b). These observations confirm the mechanism of the thrombolysis-enhancing effect of UA. Oxidative stress may inhibit alteplase-mediated thrombolysis, which is prevented by UA.

### UA–alteplase combination reduces infarct volume and inhibits haemorrhagic transformation in rats

**Figure 4:**
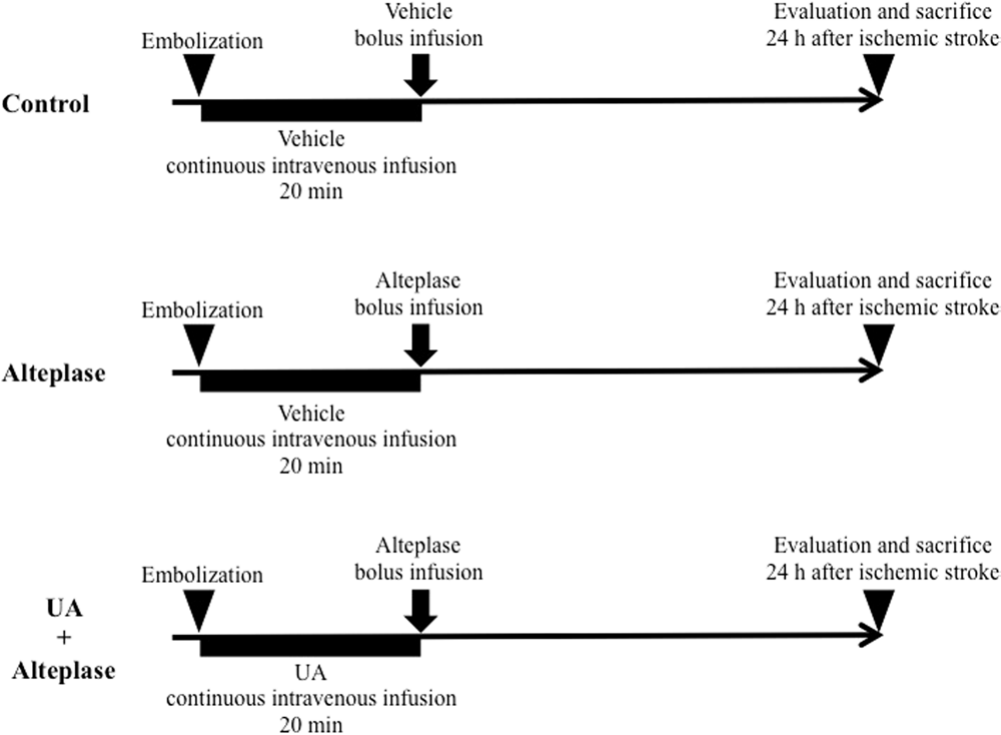
The experimental groups included the vehicle-injected control group, alteplase group, and UA–alteplase combination group. Rats that survived 24 h after establishing cerebral ischemia were euthanized (n = 7 per group). Abbreviation: UA, uric acid.

**Figure 5:**
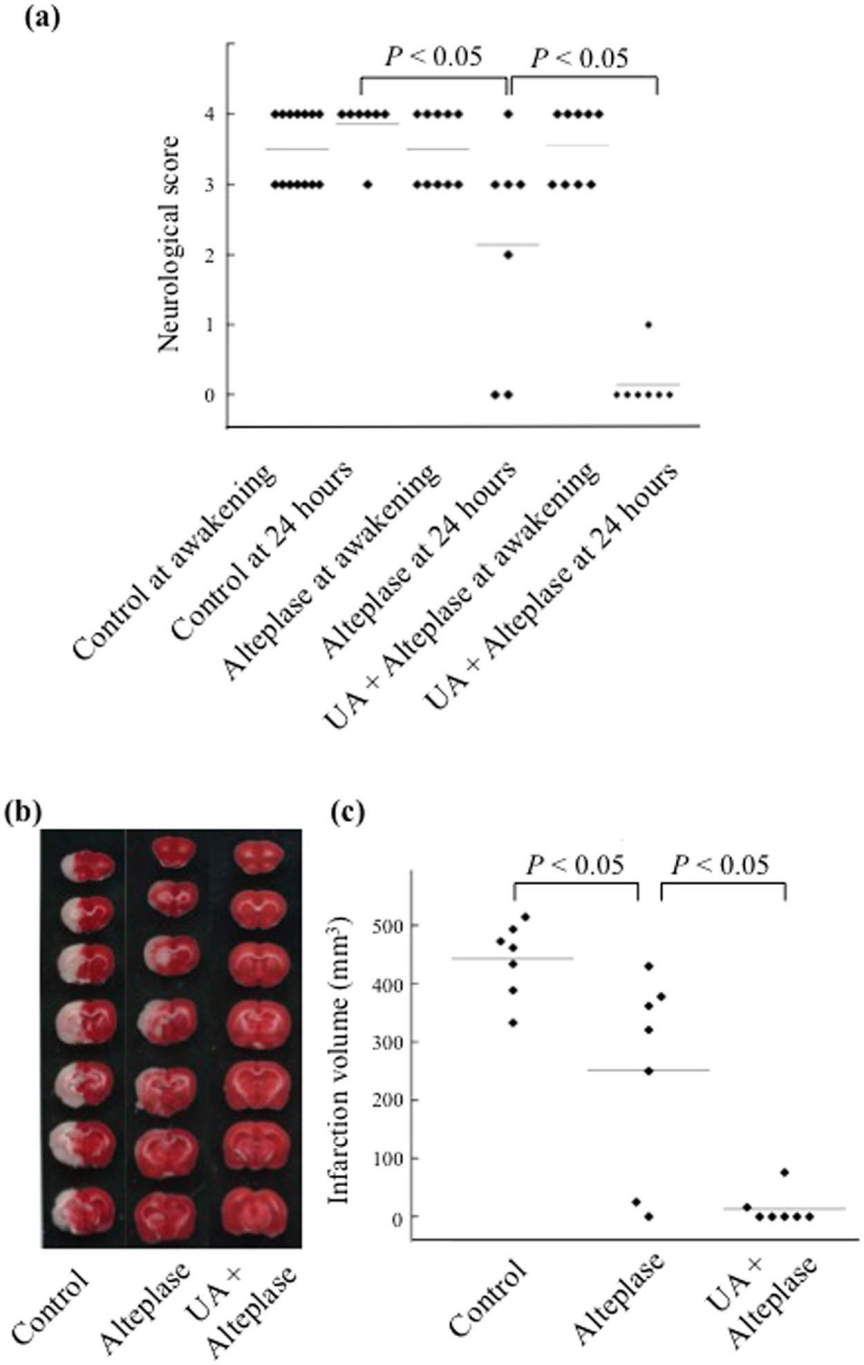
Effect of UA–alteplase combination therapy in a rat model of thromboembolic clot-induced cerebral ischemia. (**a**) Neurological score in the control, alteplase, and UA–alteplase groups. (**b**) Representative figures of 2,3,5-triphenyltetrazolium chloride-stained brain sections of rats. Normal brain tissue stains deep red, and ischemic lesions are white (unstained). (**c**) Infarct volume in the control, alteplase, and UA–alteplase groups. The horizontal lines represent the mean values. Abbreviation: UA, uric acid.

**Figure 6:**
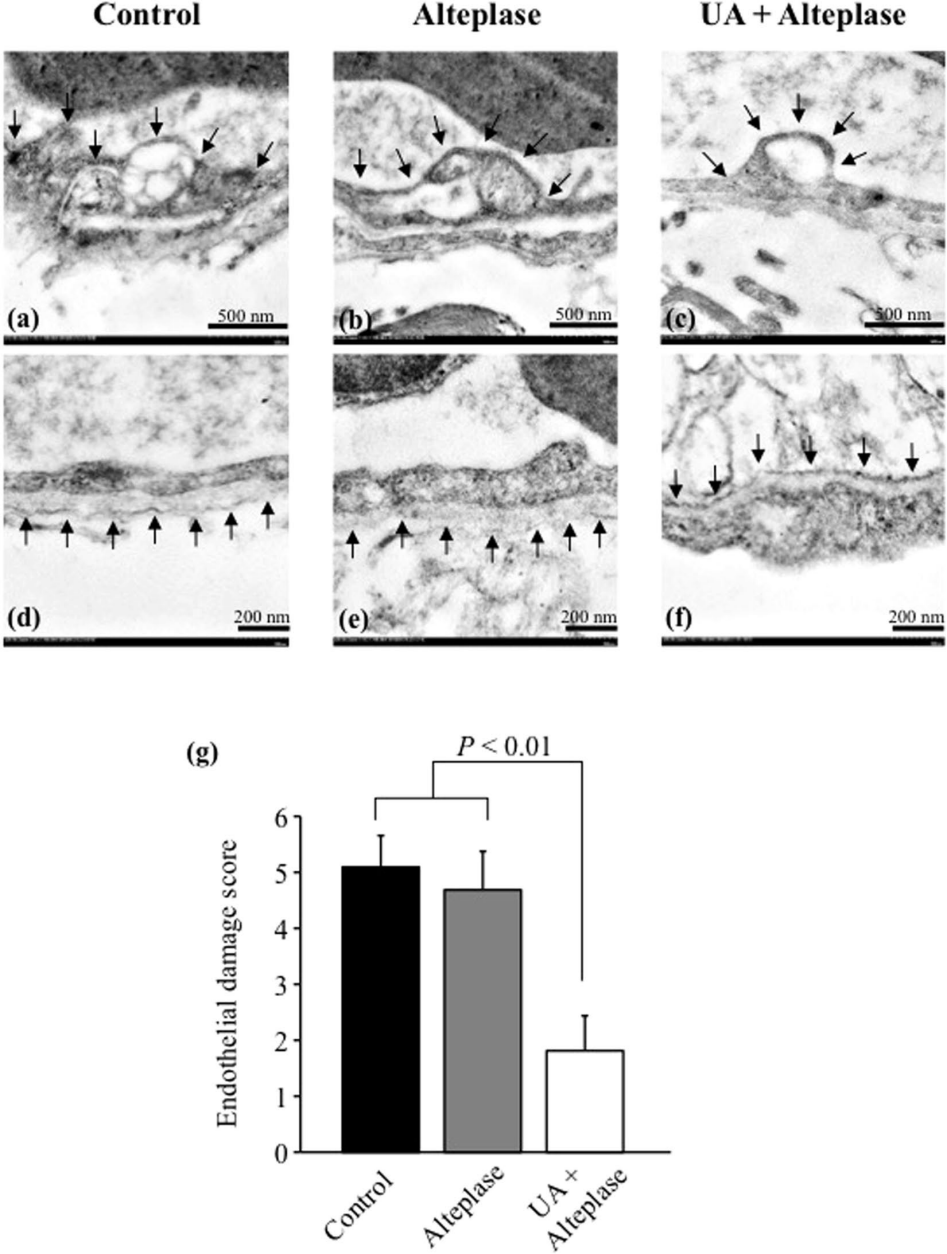
Effect of UA on alteplase-damaged vascular basement membrane by transmission electron microscopy. (**a**–**c**) Fine morphology of endothelial cells in alteplase with or without UA and control rats, respectively. Arrows indicate the severity of endothelial cell swelling, as shown by vacuolization size, and large swelling observed in alteplase without UA and control rats. (**d**–**f**) Fine morphology of the basement membranes of endothelial cells in alteplase with or without UA and control rats, respectively. Arrows indicate the lining along the vascular diameter. The basement membranes were highly disrupted in the alteplase without UA and control groups. (**g**) Endothelial damage was significantly lower in the alteplase with UA group compared with the other groups.

We evaluated the rat thromboembolic stroke model, in which the distal internal carotid artery, proximal portion of the anterior cerebral artery, middle cerebral artery, and posterior cerebral arteries were occluded by autologous thrombi (Figs 4, 5, and 6). We compared the vehicle-injected control, alteplase, and UA–alteplase combination groups, which allowed us to investigate the therapeutic effects of UA–alteplase combination therapy (Fig. 4).

We did not use a CBF monitor as previously reported^14^. The neurological score of all rats after awakening was 3 or 4, and the differences among the three groups of rats assigned to the different treatments were not statistically significant (Fig. 5a). Therefore, our method induced cerebral ischemia without using a CBF monitor.

We then evaluated the infarct volume, neuromotor function, mortality rate, haemorrhagic transformation, and adverse drug reactions at 24 h after ischemia in the three groups. Compared with the controls, alteplase significantly reduced the infarct volume (*P* < 0.05) (Fig. 4b,c). However, the infarct volume was further significantly decreased in rats receiving UA–alteplase (*P* < 0.05) (Fig. 4b,c). The neurologic score in rats receiving alteplase was significantly lower than that in the controls (*P* < 0.05) (Fig. 4d). Additionally, rats treated with UA–alteplase had significantly lower neurologic scores than the rats treated with alteplase (*P* < 0.05) (Fig. 4d). The mortality rate tended to be slightly lower in the UA–alteplase group than in the alteplase group. However, we did not find any statistically significant difference among the three groups (Table 2). The haemorrhagic transformation rate tended to be lower in the UA–alteplase group than in the alteplase group. However, like mortality rate, we found no statistically significant difference among the three groups for this measure (Table 2).

**Table 2:** Therapeutic Effects and Laboratory Data in the Rat Thromboembolic Ischemic Model.

	Control	Alteplase	UA–alteplase combination
	n (%)	n (%)	n (%)
Total number of rats	14 (100)	10 (100)	9 (100)
Rats that expired	7 (50)	3 (30)	2 (22)
Haemorrhagic transformation	0 (0)	2 (20)	0 (0)
**Blood test data**			
AST (U/L)	151.9 ± 28.6	169.4 ± 37.5	165.9 ± 61.2
ALT (U/L)	38.1 ± 10.9	40.3 ± 9.0	38.4 ± 12.6
BUN (mg/dl)	13.5 ± 2.4	14.7 ± 1.9	14.6 ± 2.6
Cr (mg/dl)	0.17 ± 0.08	0.19 ± 0.07	0.16 ± 0.05

Blood test data are shown as the mean ± standard deviation.

UA, uric acid; BUN, blood urea nitrogen; Cr, creatinine; AST, aspartate transaminase; ALT, alanine transaminase.

Adverse drug reactions, including renal and hepatic disorders, were not apparent because the serum aspartate transaminase, serum alanine transaminase, blood urea nitrogen, and serum creatinine levels were not significantly different among the three groups (Table 2). In conclusion, UA synergized with acute alteplase treatment in this experimental thromboembolic stroke model.

To determine the vascular protective effect of UA–alteplase combination therapy in ischemic brain, transmission electron microscopy was performed (Fig. 6). We found that both vascular endothelial cells and their basement membranes were better conserved in the UA–alteplase combination treated rats compared with the alteplase treated rats. Although endothelial cell swelling was also observed in UA–alteplase group (Fig. 6c), the severity of this alteration was lower than in the alteplase group (Fig. 6b). Interestingly, vascular basement membrane in the UA–alteplase group (Fig. 6f) was better conserved than in the alteplase group (Fig. 6e) with the complete lining along the vascular diameter. Our results demonstrated the efficacy of UA–alteplase combination therapy as it significantly improved the microvasculature in ischemic brains damaged by alteplase (Fig. 6g).

## Discussion

**Figure 7:**
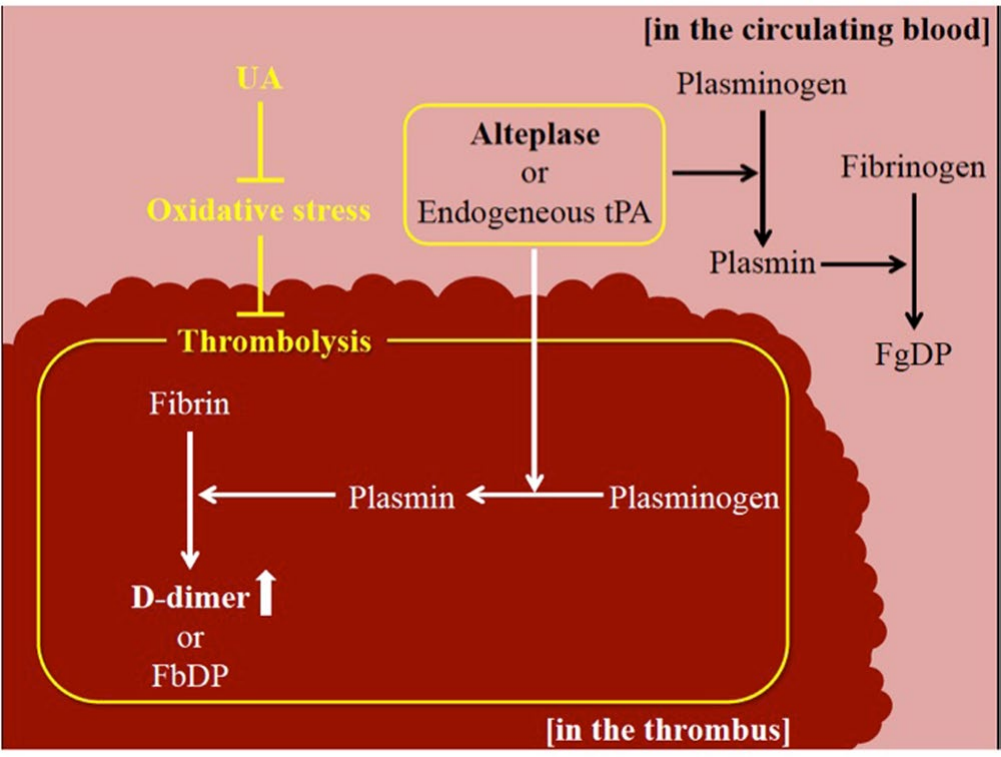
Schema showing the potential actions of UA against oxidative stress in thrombolytic pathways. Those in yellow indicate mechanisms suggested by the findings of our study. Abbreviations: UA, uric acid; tPA, tissue plasminogen activator; FbP, fibrin degradation product; FgDP, fibrinogen degradation product.

A previous *ex vivo* study reported that alteplase dissolved retrieved human cerebral thromboemboli and induced D-dimers (i.e., fibrin degradation products), minimum protein fragment^15^. However, no studies have determined whether UA enhances the induction of D-dimer release by alteplase. We found that although UA enhanced alteplase-mediated thrombolysis, UA alone did not attenuate thrombogenesis or enhance thrombolysis. This suggests that UA suppresses the inhibitory action of oxidative stress on alteplase-mediated thrombolysis (Fig. 7) in agreement with previous clinical studies^5^.

*In vitro* studies demonstrated the enhancing effect of UA against alteplase-mediated thrombolysis was confirmed in PRP, PPP, and whole blood. Whole blood contains leukocytes, unlike PRP or PPP. Moreover, H_2_O_2_ inhibited alteplase-mediated thrombolysis in whole blood samples and PPP. UA may enhance alteplase-mediated thrombolysis by inhibiting the generation of ROS by platelets, plasma components, and leukocytes. Platelets were reported to cause oxidative stress and to release platelet-derived exosomes that in turn generate oxidative stress^16^. The effect of UA that we observed in PRP and PPP samples may reflect its activity against platelet- or exosome-derived oxidative stress.

Although we were unable to identify the precise mechanism by which UA enhances the activity of alteplase, previous *in vitro* and *in vivo* studies revealed that tPA induces oxidative stress. Tissue plasminogen activators are Mac-1 (CD11b/CD18) ligands; Mac-1 mediates adhesion-dependent H_2_O_2_ production by human neutrophils^17^. Moreover, alteplase induced oxidative stress factors, such as 4-hydroxy-2-nonenal and N-(hexanoyl)-lysine (lipid peroxidation markers), 8-hydroxy-2′-deoxyguanosine (a DNA oxidation marker), and advanced glycation endproducts (protein oxidation markers) in the rat brain^18^. Therefore, the presence of an antioxidant, such as UA, may enhance the thrombolysis of alteplase.

The *in vitro* studies showed that although UA had no thrombolytic effect when administered alone, UA enhanced the thrombolytic effect of alteplase. Thus, UA–alteplase combination therapy may increase the incidence of adverse events such as intracerebral haemorrhage in patients treated for AIS. Indeed, in the URICO-ICTUS trial, symptomatic intracerebral haemorrhage did not differ significantly between the UA–alteplase (4%) and placebo–alteplase groups (3%)^19^. The fibrin-binding affinity of alteplase can be impaired by exposure to oxidative stress, and the characteristic advantage of thrombus selectivity of alteplase in spontaneous thrombolysis and thrombolytic therapy may be diminished in environments where oxidative stress levels are high in the *in vitro* study^20^. Plasminogen is present in circulating blood and thrombi, and plasmin degrades both fibrinogen and fibrin. Therefore, alteplase may activate plasminogen in the circulating blood rather than in thrombi under high oxidative stress conditions, resulting in the production of FDP from fibrinogen in the circulating blood, while fibrin in thrombi may decompose to a lesser degree. UA–alteplase combination therapy may increase the affinity of alteplase for fibrin and cause plasmin activation to be selective for thrombi. Therefore, fibrinogen degradation associated with plasminogen activation in the circulating blood, but not in thrombi, is less likely to occur. Therefore, the risk of bleeding tendencies was not increased. UA may enhance thrombolysis but not increase adverse events such as intracerebral haemorrhage because UA enhances alteplase-mediated thrombolysis, likely by preventing the inhibition of alteplase-induced fibrinolysis by oxidative stress in thrombi (Fig. 7). Transmission electron microscopy showed that UA attenuated alteplase-damaged vascular basement membranes in the *in vivo* study, which might account for the lack of increased symptomatic intracerebral haemorrhage in the URICO-ICTUS trial.

In the URICO-ICTUS trial, UA administration started after the initiation of alteplase^21^. The timing of UA administration (before, during or after alteplase administration) is crucial, given that oxidative stress may inhibit thrombolysis. It is recognized that oxidative stress is produced by reperfusion or recanalization of ischemic brains in animal models of transient ischemia^22^. Nevertheless, biomarkers of oxidative stress were already high before recanalization in patients with AIS subsequently treated with alteplase^23^, suggesting that UA should be administered promptly before alteplase. The outcomes of future large studies investigating different timings of UA administration might determine whether this combination therapy is a breakthrough treatment for AIS.

### Study Limitations

In clinical practice, alteplase is administered after thrombosis and blood vessel occlusion. In the present experiments, however, it was added before thrombosis was initiated. Furthermore, the reduction in the AUC30 in the T-TAS assay does not indicate whether thrombogenesis is being inhibited or thrombolysis is being promoted. However, the elevated D-dimer concentrations in the sump solutions suggest that thrombolysis (lysis of fibrin clots) occurred after thrombogenesis, implying that UA enhanced alteplase-mediated thrombolysis in the T-TAS assay.

In the T-TAS evaluation using whole blood and PRP samples from 6 volunteers in the current study, the individual differences in response to H_2_O_2_ were large and statistically superior data could not be obtained. Therefore, in the experiment using PPP, pooled PPP samples were used. Differences in the D-dimer levels in the PPP samples between the H_2_O_2_ + UA + alteplase group and H_2_O_2_ + alteplase group were similar (Fig. 3b). We used H_2_O_2_, a precursor/sensitizer of hydroxyl radicals, as an inducer of oxidative stress. A previous report^24^ demonstrated that UA had a high scavenging rate constant against superoxide anions and alkoxyl radicals; however, its scavenging abilities against hydroxyl radicals, alkylperoxyl radicals, methyl radicals and singlet oxygen were low when compared with other antioxidants. Therefore, future studies using various free radicals should be initiated.

The direct determination of oxidative stress such as that caused by ROS or free radicals is not easy. Moreover, obtaining high reproducibility is difficult because the circadian variation of endogenous tPA or PAI-1 is intense, and these agents are very unstable. The direct binding affinity of alteplase, H_2_O_2_, and UA against precipitated fibrin should be evaluated, but we could not perform such an evaluation using an Octet system (Pall ForteBio, Fremont, CA) by the analysis method of inter-biomolecule interaction.

This study enrolled 6 healthy young volunteers. Future studies should use samples from patients with AIS, because these samples might contain larger amounts of free radicals and the endogenous fibrinolysis system may be accelerated. Therefore, a careful analysis of the data will be required.

## Methods

The experimental animal protocol was approved by the Institutional Animal Care and Use Committee of Kagoshima University (Kagoshima, Japan) (Ethic approval number: MD16014, Approval date: 13 May 2016). The clinical study protocol was approved by the local ethics committee of Kagoshima University (Ethic approval number: 23–115, Approval date: 28 December 2011), and written informed consent was obtained from all individuals prior to their participation.

### Human Blood Samples

Blood samples from six healthy, fasting Japanese volunteers (4 males, 2 females; mean age, 29.8 ± 7.4 years) were collected in plastic tubes containing 3.2% sodium citrate (Terumo Co.). None of the volunteers had taken antithrombotic drugs within 2 weeks of the study. Normal ranges for the T-TAS analysis have not yet been defined, but the T-TAS findings of all volunteers’ samples lay within 95% of the median of 123 healthy Japanese individuals who participated in our preliminary study (data not shown). Platelet-rich plasma was prepared by centrifugation at 800 rpm for 15 min, and platelet-poor plasma (PPP) was prepared by centrifugation at 3000 rpm for 15 min.

In the experiments using whole blood and PRP samples, we selected the final concentration of alteplase (500 IU/ml) based on half the maximum concentration after administration to Japanese patients with AIS^14^. Patients in the UA group received 1000 mg of UA in the URICO-ICTUS trial^5^. The peak blood concentration of UA (1000 mg) was approximately 10 mg/dL in the URICO-ICTUS pilot study^25^. Therefore, in experiments using whole blood, PRP and PPP samples, we selected 10 mg/dL of UA as the final concentration, based on a preliminary experiment and previous reports. The UA solution was prepared by dissolving 20 mg UA in a 1 mL solution containing 5% mannitol and 0.1% lithium carbonate as previously described^25^. Human blood samples include endogenous tPA and UA. In our *in vitro* studies, it is unclear whether endogenous tPA and UA had physiological activity. However, the final concentration exceeded the added concentration. In the experiments using PPP samples, we selected the final concentration of alteplase (100 IU/ml) based on the findings of a preliminary experiment^14^. The final concentration of H_2_O_2_ (100 µM) (Sigma-Aldrich Japan, Tokyo, Japan) was based on a previous report^14^, recognizing that it is difficult to estimate the local concentration of reactive oxygen species (ROS) around intravascular thrombi during AIS^26^. The total volume of the agents or vehicle in the blood sample was 5%. Therefore, in all the groups, the blood sample volume was always 95%.

### T-TAS

The thrombolytic effects of alteplase, UA, and the combination of alteplase and UA were compared with the controls under flow conditions using T-TAS in whole blood and PRP. To quantify thrombogenesis and thrombolysis under flow conditions, the T-TAS assay was performed as previously described^13^. Thrombogenesis and thrombolysis were observed in the microchip using a built-in light microscope. UA was added to the blood samples 10 min before the addition of alteplase. Hydrogen peroxide was added immediately after the addition of alteplase. As soon as alteplase or H_2_O_2_ had been administered, each sample was perfused over a microchip coated with collagen and tissue thromboplastin to promote thrombosis at a flow rate of 4 µl/min, corresponding to an initial wall shear rate of 240 per second.

### Measurement of D-Dimer Concentration in Sump Solutions of T-TAS

T-TAS sump solutions were prepared by diluting the analysed pooled PRP samples (n = 6) at a 1:25 ratio in ethylenediaminetetraacetic acid followed by centrifugation at 800 rpm for 15 min. The concentration of D-dimers was also measured in the sump solution using an LPIA-NV7 instrument and RM73-752YLK solution (LSI Medience Corporation, Tokyo, Japan).

### Measurement of D-Dimer Concentration in Human PPP Samples

D-dimers were measured to determine whether UA attenuates the inhibition of alteplase-induced fibrinolysis by H_2_O_2_. Four-hundred-microliter aliquots of 30% human PPP pooled from all 6 volunteers and TBSTC (8 mM Tris at pH 7.4, 0.008% Tween-20, and 12 mM calcium chloride) were prepared. After incubation at 37 °C for 15 min, H_2_O_2_ (100 µM), UA (10 mg/dL), or vehicle was added. After incubation at 37 °C for 10 min, alteplase (100 IU/ml) or vehicle was added. After fibrin deposition had occurred by incubating at 37 °C for 10 min, aprotinin (400 KIU/ml) was added. After centrifugation at maximum speed for 5 min, the concentration of D-dimers was measured using an ACL TOP automated analyser (Instrumentation Laboratory, Bedford, MA) (n = 5).

### Experimental Animal Model

Thromboembolic ischemia of the middle and posterior cerebral arteries was induced by homologous blood clot in 8-week-old male Sprague–Dawley rats (KBT Oriental Co., LTD, Tosu, Japan) weighing 290 to 310 g as previously described by our group^14^. Anaesthesia was induced and maintained with 2.5% to 3.0% isoflurane inhalation. The left internal carotid artery was isolated, a 24-gauge catheter (SURFLO Flash®; Terumo Co.) was inserted in the internal carotid artery, and a 5-mm clot (volume of 3.6 mm^3^) was pushed into the artery via the catheter^14^. The catheter containing the clot was hooked up to a syringe containing physiological saline. Therefore, the clot together with physiological saline was definitely inserted into the distal internal carotid artery, the proximal portion of the anterior cerebral artery, the middle cerebral artery, and the posterior cerebral artery. Consequently, all rats had neurological scores of either 3 or 4 (Fig. 5a).

We did not use a cerebral blood flow (CBF) monitor as previously described^14^. Shimamura *et al*.^27^ reported that a CBF monitor is not indispensable for this model because other surgical manipulations can be performed to establish whether brain injury and/or changes in intracranial pressure have been avoided. Additionally, dissection of the temporal muscle causes masticatory dysfunction, leading to inadequate nutrition. Instead, we used only rats with a neurological score of 3 or 4 after awakening. The rats were evaluated for neurological deficits after awakening and at 24 h after thromboembolism. A neurological grading system with a 5-point motor function scale (0–4) was used as previously described^26^. The scale was as follows: 0 = no apparent deficits, 1 = right forelimb flexion, 2 = decreased right forelimb grip when tail is pulled, 3 = spontaneous movement in all directions with right circling only when tail is pulled, and 4 = spontaneous right circling. The occurrence of death was recorded and included near death or severe epileptic seizure that precluded neurological tests.

Twenty-four hours after thromboembolism, three groups (n = 7 per group) of surviving rats were studied: the vehicle-injected control group, alteplase group, and UA–alteplase group. Because a similar experiment was previously reported^6^, the animal experiments were performed using a minimal sample size of 7 rats per group as previously reported^28,29^. According to the IMPROVE (Ischemia Models: Procedural Refinements Of *In vivo* Experiments)^30^ guidelines, we used a randomisation protocol to ensure that each cage contained rats from different groups, including control, alteplase, and UA-alteplase. Similarly, to rule out cage effects, animals within each cage were randomised to different groups. Finally, after inducing cerebral ischemia, rats were randomly assigned to the groups. Rats in the UA–alteplase group received the following procedure. Immediately after thromboembolism, UA (Wako Pure Chemical Industries, Ltd., Osaka, Japan) (16 mg/kg body weight) was administered over a 20-min period via a jugular vein catheter using an infusion pump^6,15^. At 20 min after thromboembolism, alteplase (Mitsubishi Tanabe Pharma Corporation, Osaka, Japan) (3 mg/kg body weight) was administered as previously described^14^. The alteplase group received vehicle instead of UA, followed by alteplase treatment^15^. The control group received injections of vehicle instead of UA and alteplase. The timing and rate of administration of UA and alteplase were determined via preliminary experiments to maximize the effect of UA. Therefore, the dose of alteplase was lower than that in previous reports using 10 mg/kg^31,32^.

### Rat Blood Samples and Histology

Twenty-four hours after thromboembolism, the rats were deeply anesthetized via an intraperitoneal injection of 4% chloral hydrate (10 ml/kg). Blood samples were collected from the axillary vein. To evaluate adverse drug reactions including renal and hepatic disorders, the serum aspartate transaminase, serum alanine transaminase, blood urea nitrogen, and serum creatinine levels were measured by an enzymatic method using a Fuji DRI-CHEM Slide Kit (Fujifilm Medical, Tokyo, Japan)^14^.

Immediately after venous blood sampling, the rats were killed and their brains excised 24 h after thromboembolism as previously described^14^. Physiological saline was transcardially perfused before decapitation. The brain was carefully removed and cut into six 2-mm-thick coronal sections from the frontal tip using a brain slicer. The slices were then immersed in a 1% solution of 2,3,5-triphenyltetrazolium chloride in phosphate buffered saline (pH 7.4) at 37 °C for 10 min. After staining, the sections were scanned to determine the ischemic infarct volume. The infarctions were measured using Scion Image software v. Beta 4.0.3 (Scion Corp., Frederick, MD). The total infarct area (mm^3^) was multiplied by the thickness of the brain sections to obtain the infarct volume. Additionally, the presence of visible haematomas or haemorrhagic transformation was recorded.

For transmission electron microscopy (alteplase group, n = 3; UA–alteplase group, n = 3), brains were collected from an ischemic area in each experimental group and immediately fixed with 2.5% glutaraldehyde in 0.1 M sucrose phosphate buffer (SPB) for 1 h, then fixed with 1% osmium tetroxide for 1 h. The tissues were dehydrated, infiltrated, and embedded in LR white resin (EMS, Hatfield, PA). Embedded tissues were polymerized in a 65 °C incubator for 48 h. All blocks were cut to 90–100 nm thickness and stained with uranyl acetate and lead citrate. Brain ultrastructure was examined by transmission electron microscopy (Hitachi; model HT7700, Japan) focusing on blood brain barrier integrity characterized by endothelial cell swelling and basement membrane disruption. Changes in the fine structures were qualitatively scored by grading from 0 to 3 as follows: 0; no alterations, and 1–3; mild (<25% present in the vascular diameter), moderate (25–50% present in the vascular diameter), or severe (>50% present in the vascular diameter) swollen or discontinued membranes, respectively. Scoring of the endothelial damage severity (0–6) was then calculated by combining the scores for endothelial cell swelling and basement membrane disruption.

### Statistical Analysis

Measurement and quantification of data were performed by investigators blinded to the treatments. The neurological score, infarction volume, and blood test data were analysed using the Steel–Dwass method or Bonferroni–Dunn method as appropriate for multiple comparisons. The mortality and haemorrhagic transformation rates were compared by an *m* × *n* χ^2^ test. The area under the curve at 30 min (AUC30) was calculated to evaluate the extent of thrombogenesis or thrombolysis as previously described^13^. Comparison between two groups was performed using the paired *t*-test or Wilcoxon’s signed rank test as appropriate. Data are presented as the mean ± standard deviation unless otherwise indicated. Differences of *P* < 0.05 were considered statistically significant. All analyses were performed using SPSS Statistics (version 20; IBM Corp., Armonk, NY).

## Electronic supplementary material

Supplementary Movie 1
